# ADVERSE CHILDHOOD EXPERIENCES AND MENTAL HEALTH IN MIDDLE AGE AND OLDER ADULTS: THE MEDIATIONAL EFFECT OF RESILIENCE

**DOI:** 10.1093/geroni/igae098.3426

**Published:** 2024-12-31

**Authors:** Christopher Griffith, Tiana Broen, Julia Scott, Leilani Feliciano

**Affiliations:** University of Colorado Colorado Springs, Colorado Springs, Colorado, United States; University of Colorado Colorado Springs, Colorado Springs, Colorado, United States; University of Colorado Colorado Springs, Colorado Springs, Colorado, United States; University of Colorado Colorado Springs, Colorado Springs, Colorado, United States

## Abstract

Adverse childhood experiences (ACEs), physical health problems, and poor health perception are known predictors of subsequent poor mental health later in life. To support aging adults in their mental wellbeing, it is crucial to understand how they may recruit internal resources, such as resilience, to cope with past adverse experiences and current health difficulties. The current study used structural equation modeling (SEM) to examine the mediational effect of resilience on the pathways between adverse childhood experiences, sociodemographic variables, and health-related variables to mental wellbeing (a latent factor represented by depression, anxiety, and stress scores, higher score = lower mental wellbeing). Survey data were analyzed from a sample of adults aged 40 to 94 years (N = 334; Mage = 60.46; 48.8% women; 86.2% white). Number of ACEs were significantly correlated with age (β = -8.86, p <.001), number of physical health conditions (β = 0.44, p <.05) and mental health conditions (β = 1.11, p <.001), and poorer subjective health perception (β = 0.47, p <.001). Resilience mediated the pathways from subjective health perception to poor mental wellbeing (β =.05, p <.01), number of mental health conditions to poor mental wellbeing (β =.11, p <.01), and number of ACEs to poor mental wellbeing (β = 0.03, p <.05). Overall, these results highlight the significance of ACEs on mental wellbeing outcomes, and the importance of enhancing resilience in older clients with a history of ACEs.

